# A feasibility study of a theory-based intervention to improve appropriate polypharmacy for older people in primary care

**DOI:** 10.1186/s40814-017-0166-3

**Published:** 2017-07-20

**Authors:** Cathal A. Cadogan, Cristín Ryan, Gerard J. Gormley, Jill J. Francis, Peter Passmore, Ngaire Kerse, Carmel M. Hughes

**Affiliations:** 1 School of Pharmacy, Royal College of Surgeons in Ireland, Dublin, Ireland; 2 Department of General Practice, Queen’s University Belfast, Belfast, UK; 3 School of Health Sciences, City University of London, London, UK; 4 Centre for Public Health, Queen’s University Belfast, Belfast, UK; 5 School of Population Health, University of Auckland, Auckland, New Zealand; 6 School of Pharmacy, Queen’s University Belfast, 97 Lisburn Road, Belfast, BT9 7BL UK

**Keywords:** Polypharmacy, Intervention, Feasibility, Behaviour change, Prescribing, Theoretical Domains Framework, General practice

## Abstract

**Background:**

A general practitioner (GP)-targeted intervention aimed at improving the prescribing of appropriate polypharmacy for older people was previously developed using a systematic, theory-based approach based on the UK Medical Research Council’s complex intervention framework. The primary intervention component comprised a video demonstration of a GP prescribing appropriate polypharmacy during a consultation with an older patient. The video was delivered to GPs online and included feedback emphasising the positive outcomes of performing the behaviour. As a complementary intervention component, patients were invited to scheduled medication review consultations with GPs. This study aimed to test the feasibility of the intervention and study procedures (recruitment, data collection).

**Methods:**

GPs from two general practices were given access to the video, and reception staff scheduled consultations with older patients receiving polypharmacy (≥4 medicines). Primary feasibility study outcomes were the usability and acceptability of the intervention to GPs. Feedback was collected from GP and patient participants using structured questionnaires. Clinical data were also extracted from recruited patients’ medical records (baseline and 1 month post-consultation). The feasibility of applying validated assessment of prescribing appropriateness (STOPP/START criteria, Medication Appropriateness Index) and medication regimen complexity (Medication Regimen Complexity Index) to these data was investigated. Data analysis was descriptive, providing an overview of participants’ feedback and clinical assessment findings.

**Results:**

Four GPs and ten patients were recruited across two practices. The intervention was considered usable and acceptable by GPs. Some reservations were expressed by GPs as to whether the video truly reflected resource and time pressures encountered in the general practice working environment. Patient feedback on the scheduled consultations was positive. Patients welcomed the opportunity to have their medications reviewed. Due to the short time to follow-up and a lack of detailed clinical information in patient records, it was not feasible to detect any prescribing changes or to apply the assessment tools to patients’ clinical data.

**Conclusion:**

The findings will help to further refine the intervention and study procedures (including time to follow-up) which will be tested in a randomised pilot study that will inform the design of a definitive trial to evaluate the intervention’s effectiveness.

**Trial registration:**

ISRCTN18176245

**Electronic supplementary material:**

The online version of this article (doi:10.1186/s40814-017-0166-3) contains supplementary material, which is available to authorized users.

## Background

Older people are the greatest consumers of healthcare resources in developed countries. Given the predicted increases in the size of the older population, the use of medicines in this population cohort has been described as the ‘single most important health care intervention in the industrialised world’ [1]. The prescribing of multiple medicines, also termed polypharmacy, is increasingly common in older people [2, 3] and is considered to be one of the most pressing prescribing challenges [4]. Ensuring ‘appropriate polypharmacy’ in this patient cohort, whereby prescribing is evidence-based and reflects patients’ clinical needs, is a challenge faced by clinicians that is of considerable clinical and economic importance [5]. Polypharmacy is the principal contributing factor to potentially inappropriate prescribing in older populations [6, 7] and has been associated with a range of negative clinical consequences, including adverse drug events (ADEs) and medication non-adherence [8]. Potentially inappropriate prescribing in older people places a substantial financial burden on health services [6, 7].

Improving appropriate polypharmacy in older people poses challenges on a number of levels. For example, the availability of guidelines to inform prescribing practices/decisions for older people who commonly suffer from more than one chronic condition (i.e. multimorbidity) is currently lacking [9]. Prescribing guidelines typically deal with single diseases, and when applied to complex multimorbid patients, they often fail to provide guidance on how to prioritise treatment recommendations and can act as a contributing factor for polypharmacy [10]. There has been some progress in addressing this issue with the recent publication of guidelines for the clinical assessment and management of patients with multimorbidity by the National Institute for Health and Care Excellence (NICE) [11]. However, it will be some time before the clinical impact of these guidelines is fully established, particularly as their implementation may require some reorganisation in the way in which care is currently delivered [12].

Another challenge is that there are considerable deficits in the current evidence base for interventions that target clinical practice and aim to improve outcomes for patients with complex clinical needs, such as multimorbidity and polypharmacy [13, 14]. For example, a Cochrane systematic review of interventions to improve appropriate polypharmacy in older people found that the quality of available evidence was low, owing to risk of bias in the included studies. In addition, details of intervention development and delivery were lacking in published reports [14]. This prevents researchers and clinicians from understanding how the interventions were intended to exert their effects and limits the potential for effective interventions to be replicated and optimised in clinical practice. Accordingly, a more systematic approach, incorporating both evidence and theory, was recommended for the development of future interventions [14]. This is in line with the UK Medical Research Council’s (MRC) complex intervention framework, which recommends that intervention development be guided by best available evidence and appropriate theory [15]. It is recommended that after the intervention is developed, preliminary evaluations in the form of feasibility and pilot studies be conducted to estimate parameters (e.g. recruitment/retention rates, sample size) that are important for a full-scale clinical trial.

As part of a multiphase mixed methods research project, an intervention-targeting general practitioners (GPs) was developed to improve the prescribing of appropriate polypharmacy for older people (outlined below). Full details of the preliminary work underpinning the intervention’s development are reported in two related papers [16, 17]. Briefly, the intervention development process followed a systematic approach based on the MRC framework by incorporating evidence and theory [15]. A Cochrane systematic review was updated to establish the existing evidence base [14]. Qualitative interviews that were underpinned by a theoretical framework of behaviour change (i.e. Theoretical Domains Framework (TDF) [18]) were conducted with GPs. The TDF-based interviews provided a method for identifying theoretical domains that were perceived as barriers and facilitators to the prescribing of appropriate polypharmacy for older people [16]. These theoretical domains were then mapped to behaviour change techniques (BCTs) from an established taxonomy [19] and embedded in the intervention as the proposed ‘active ingredients’ [17]. Hence, a theory base was incorporated into the intervention development process, as advocated by the MRC framework [15].

This study sought to test the feasibility of this intervention, which targeted GPs to improve the prescribing of appropriate polypharmacy for older people in primary care. This involved assessing the intervention’s usability and acceptability to determine if the intervention’s content and delivery required further refinement. Recruitment methods, data collection procedures and the selection of assessment measures were also examined to determine if the study protocol required further refinement before progressing to a randomised pilot study.

## Methods

This feasibility study was conducted in two general practices. Ethical approval was granted by the Office of Research Ethics Committees Northern Ireland in advance of the study (REC reference 15/NI/0104). The study is registered with the ISRCTN registry (ISRCTN18176245).

### Aim and objectives

The primary aim of the study was to assess the feasibility of a GP-targeted intervention to improve the prescribing of appropriate polypharmacy for older people in primary care. This aim was met through the study objectives, which were to:

1. Evaluate the methods of participant recruitment
2. Assess the intervention’s usability and acceptability (to GPs and patients)
3. Assess the fidelity of delivery of pre-specified behaviour change techniques that were embedded in the intervention
4. Evaluate data collection procedures (including the utility of selected assessment measures/tools)

### Sampling and recruitment

A convenience sampling method was used to recruit GPs from two general practices (one urban and one rural) into the study. Practices were sampled from nine general practices that had participated in the earlier qualitative interview phase of the project (see Cadogan et al. [16]). The main reason for approaching these practices initially was because they had participated in the earlier phase of the study during which the intervention had been developed to target specific theoretical domains that were reported to be affecting the prescribing of appropriate polypharmacy by GPs within these practices. Therefore, these GPs were considered to be best placed to test the usability and acceptability of the intervention in addressing the specific challenges that they (and/or their colleagues) reportedly faced in clinical practice.

The researcher (CC) approached two practices and invited GPs within the practice to participate in the feasibility study. General practices were contacted until two practices had agreed to hold a meeting with the researcher with a view to participating in the study. At these meetings, the researcher provided GPs with an overview of the feasibility study and intervention (described below), as well as a study folder containing all relevant study documentation (i.e. participant information leaflets, consent forms, written instructions and login details for accessing the intervention’s online video component (described below). A maximum of five GP participants were sought per practice so that the study workload could be divided evenly across GPs within recruited practices (i.e. each GP would engage with one patient participant as part of the study).

Each participating general practice recruited five older patients meeting inclusion criteria (i.e. over the age of 65, receiving four or more regular medicines, not cognitively impaired, resident in the community) into the study. Nursing home residents were excluded from the study as their clinical complexity and context is very different to community-dwelling patients. Patient recruitment was facilitated by nurses from the Northern Ireland Clinical Research Network (NICRN) who screened practice records and issued written invitation letters and study information sheets to patients meeting inclusion criteria. GPs reviewed the final list of identified patients before invitation letters were issued to ensure that patients met the inclusion criteria and were suitable for review. The invitation letters asked patients to contact the practice if they were interested in having a consultation with their GP about their medicines on a specified date (practice 1: August 2015, practice 2: September 2015). GPs agreed to accommodate any patients who were interested in booking a consultation but were not able to attend the practice on the specified date. However, these patients were not recruited into the study. Invitation letters were issued in batches and the number of letters issued in the first round was agreed between the NICRN nurses and the participating GPs in order to ensure that the practice could accommodate patients if uptake was higher than anticipated. This resulted in an initial batch of 20 and 15 invitation letters being issued from practices 1 and 2, respectively. The patient sampling quota was reached in each practice after the first batch of invitation letters was issued.

The researcher obtained written informed consent from all GP and patient participants prior to consultations taking place. Each general practice was offered financial compensation (£500) for the time and resources associated with study participation. GP participants were provided with a certificate of participation for their continuing professional development portfolios. No incentive was offered to patient participants.

### Intervention content and delivery

The intervention consisted of a short online video (approximately 11.5 min) that was delivered to GPs and demonstrated how GPs prescribe appropriate polypharmacy during a typical consultation with an older patient (BCT: ‘Modelling or demonstrating of behaviour’—as defined according to BCT Taxonomy Version 1 [19]). The video was scripted by the research team, and the clinical content was developed by an academic GP and geriatrician with input from pharmacists. The video included feedback from both a practising GP and a simulated patient emphasising the positive outcomes of the consultation (BCT: ‘Salience of consequences’—as defined according to BCT Taxonomy Version 1 [19]). A professional video production team was commissioned to record the video which was delivered to GP participants online. The video was uploaded onto a secure web server using the ‘Riverside®’ software programme and accessed by GPs using a generic username and password. Written instructions and login details to access the video were included in the study folder that each practice received from the researcher. Patient participants were not provided with access to the video.

As a complementary intervention component, patients were invited to scheduled medication review consultations with GPs. Explicit plans were made at weekly meetings with practice staff (i.e. GPs, practice nurses, reception staff) of when and how GPs would ensure that target patients were prescribed appropriate polypharmacy (BCT: ‘Action planning’—as defined according to BCT Taxonomy Version 1 [19]). GPs had oversight of the patients who were being invited to participate in the study and allocated time slots in their weekly schedule to conduct review consultations with patients as part of the study. Reception staff scheduled the consultations for patients who responded to the invitation letter and prompted GPs to carry out the agreed plan to review target patients’ medications when they presented at the practice by notifying participating GPs that the patients were attending a scheduled consultation as part of the study (BCT: ‘Prompts/cues’—as defined according to BCT Taxonomy Version 1 [19]).

#### Specification of intervention content using BCTs

A BCT coding exercise was conducted by way of a fidelity check in order to investigate if the pre-specified BCTs (i.e. ‘Modelling or demonstrating of behaviour’, ‘Salience of consequences’) embedded in the video were readily identifiable to an independent group of researchers. This was intended to add to the methodological rigour of the intervention development process that has previously been described (see Cadogan et al. [17]).

The video coding exercise was performed by an independent group of seven researchers who had previously undergone standardised training in BCT coding (through either tutorials or an online course). Coders had no prior involvement in the project. The group was shown the video, and each member was provided with a transcript of the video (i.e. the script that was used by the actors and descriptive details of each scene). Each coder independently coded the video content by assigning BCTs from the published BCT taxonomy [19] to sections of the video transcript. Coders also reported ratings of their confidence in each coding judgement using a rating scale from the standardised BCT training programme (‘+’ = fairly confident; ‘++’ = extremely confident) [20]. Coders were instructed to code sections of the video transcript to any given BCT once only, even if the particular BCT was deemed to have been delivered multiple times. A BCT was deemed to have been embedded in the video where the majority of coders (≥4) had assigned the BCT to sections of the video transcript.

### Outcomes

The primary feasibility outcomes were the usability and acceptability of the intervention to GPs. Feedback from patients regarding the acceptability of the medication review with their GP was also included in the overall evaluation.

As secondary feasibility outcomes, study parameters were investigated that would ultimately help to inform the design of a future pilot study (i.e. recruitment, data collection procedures). For example, the study assessed the feasibility of collecting feedback from GPs and patients using self-administered questionnaires that were to be completed in a hard copy form and returned using postage-paid envelopes. The study also assessed the feasibility of applying validated assessment tools to the patients’ medical record data, as well as the utility of selected assessment tools.

### Data collection and analysis

In assessing the feasibility study outcomes, data were collected from GPs, patients and practice records as described below.

#### Feedback from GP and patient participants

Recruited GPs were asked to complete a structured paper-based questionnaire (Additional file 1) after they had watched the online video and performed medication reviews with recruited patients. The questionnaire assessed the intervention’s usability and acceptability. We were unable to identify any previously validated tool that would be directly applicable to the assessment of our intervention. We had originally intended to include the System Usability Scale (SUS) in the GP feedback questionnaire [21]. SUS is a simple 10-item scale designed to provide a subjective assessment of a system’s usability. However, as the scale’s questions related more specifically to the video’s online mode of delivery as opposed to its content, it was not considered to be an appropriate tool for the purpose of the current feasibility study. Instead, we adapted items that were included in the GP feedback questionnaire of the online video (i.e. a form of technology) from Davis’ Perceived Usefulness Scale [22]. This scale forms part of the Technology Acceptance Model (TAM) which was developed to explain technology usage behaviour [22].

Feedback was gathered under three main subheadings: ‘Video use and usefulness’, ‘Online system for accessing video’ and ‘Medication review process’ (Table 1). Additional questions were included to investigate whether GPs would recommend any changes to the video or online delivery system for future studies. The questionnaire included fixed category response options and 5-point Likert scales. Free-text response options were also provided to allow respondents to elaborate on their responses.

**Table 1:** Sample questionnaire items

Questionnaire section	Questionnaire item
Video use and usefulness	Using videos like this would make it easier for me to perform medication reviews with older patients in daily practice. Using videos like this would increase the number of medication reviews that I perform with older patients in daily practice.
Online system for accessing video	Did you experience any problems using the online system? (If yes, please briefly outline) If the online system was to be further developed as a resource to assist GPs in prescribing appropriate polypharmacy for older people, what types of additional material and/or resources would you like to see included?
Medication review process	Think back to a medication review that you performed with an older patient as part of this study. Did you make any changes to the patient’s prescription? If yes, was there anything that made it difficult for you in putting this change(s) into effect? (please briefly outline)

Recruited patients were asked to complete a feedback questionnaire (Additional file 2) after the consultation with their GP. The questionnaire examined patients’ views and experiences of the medication review process under two main subheadings: ‘medication reviews with the GP’ and ‘the scheduled consultation’. The questionnaire included the same types of response options as the GP questionnaire.

All questionnaires were completed in hard copy form and returned to the researcher (CC) using a pre-paid postage envelope labelled with the researcher’s work address. Questionnaire data were analysed using IBM SPSS Statistics v.21. Simple descriptive analyses were conducted to summarise the data. Content analysis was performed on free-text questionnaire responses for generation of representative themes. Each of the answers to the open-ended questions was reviewed separately, and key subthemes relating to the answers were identified by the researcher (CC). A summary document that included all free-text responses and an overview of the content analysis was presented to other members of the research team for discussion. There were no disagreements with the presented results.

#### Practice record data

Clinical data were extracted from recruited patients’ medical records by research nurses from the NICRN at baseline (date of scheduled consultation) and at follow-up (1 month post-consultation). Extracted data consisted of patients’ demographics (i.e. age, gender), clinical conditions and prescribed medications (acute list and repeat list items). Validated assessments of prescribing appropriateness (i.e. Screening Tool of Older People’s potentially inappropriate Prescriptions (STOPP)/Screening Tool to Alert doctors to Right Treatment (START) criteria [23], Medication Appropriateness Index (MAI) [24]) and prescribing regimen complexity (i.e. Medication Regimen Complexity Index (MRCI) [25]) were applied to the extracted clinical data.

As there is currently no ‘core outcome set’ (i.e. an agreed or standardised set of outcomes that should be assessed and reported as a minimum) for trial evaluations of interventions aimed at improving appropriate polypharmacy [26], each assessment tool was purposefully selected by the research team. Firstly, due to the lack of a validated measure of appropriate polypharmacy, we used validated general measures of prescribing appropriateness as surrogate measures. Specifically, an implicit (judgement-based) tool and an explicit (criterion-based) tool were used (i.e. MAI [24] and STOPP/START criteria [23], respectively). This combination of tools was intended to help overcome inherent limitations of each assessment tool, details of which have been documented extensively in the literature (see Kaufman et al. [27] for a detailed overview of available prescribing tools). Assessments of prescribing appropriateness using the MAI [24] involve applying a score to each medication, and a total MAI score per person is then reported by adding the scores for individual medications. Using this approach, the possible range of scores is 3 (more appropriate) to 18 (less appropriate) for each prescribed medication. Assessments of prescribing appropriateness involving STOPP/START criteria [23] report the prevalence of each criterion within the sample population. These criteria consist of 80 STOPP criteria and 34 START criteria.

We also sought to evaluate additional factors (e.g. dosage forms, dosing frequencies), other than the number of medicines prescribed, that contribute to the overall complexity of drug regimens using the MRCI [25]. This tool involves assigning a score to quantify complexity of medication regimens based on the prescribed dosage forms, dosing frequencies and additional directions. Each assessment tool was applied by two members of the research team working independently (CC, CR). Any disagreements were resolved by consensus discussion and consultation with a third member of the research team (CH).

## Results

### Participant recruitment

Each of the first two general practices that were contacted about the study agreed to take part in the feasibility study. Four GPs (3 female, 1 male) were recruited into the study from these practices (practice 1: three GPs; practice 2: one GP). Patients who received an invitation letter and were interested in attending the practice to have a consultation with their GP about their medicines on the specified date contacted the practice. Each practice scheduled consultations between GP participants and five older patients who were receiving polypharmacy.

Each patient attended the practice for a scheduled consultation and was recruited into the study (total sample size of 10 patients; five per practice). Six of the ten patient participants were female (Table 2). Participants’ median age was 73.5 years (range 68–78 years). All patient participants were multimorbid (median number of chronic conditions: 3) and were receiving at least four regular medicines (median number of medicines: 6) at baseline.

**Table 2:** Summary statistics of patient demographics

Gender	
Male	4
Female	6
Age (years)	
Mean (±standard deviation)	73.1 (±4.04)
Median	73.5
Range	68–78
Number of repeat medications	
Mean (±standard deviation)	6.4 (±2.2)
Median	6
Range	4–10
Number of chronic conditions^a^	
Mean (±standard deviation)	3.4 (±1.4)
Median	3
Range	2–7

^a^Assessment based on clinical conditions recorded in patients’ medical records

### GP feedback

Responses to the feedback questionnaire were received from both participating practices, with three of the four GP participants returning completed questionnaires. All of the questionnaire respondents were female. The number of years respondents had been practising as GPs varied (range 9–20 years).

#### Video use and usefulness

Each GP reported watching the online video (primary intervention component) at least once prior to the first patient consultation that was scheduled as part of the study (one GP reported watching the video once and two GPs reported watching the video twice). The respondents’ views on the perceived usefulness of the video varied (Table 3). The respondents were undecided (2 neutral, 1 agree) as to whether the video would make it easier for them to perform medication reviews with older patients in daily practice.

**Table 3:** GP respondents’ views on the perceived usefulness of the video (*n* = 3)

Using videos like this would…	Strongly disagree	Disagree	Neutral	Agree	Strongly agree
…make it easier for me to perform medication reviews with older patients in daily practice.			2	1	
…improve my performance of medication reviews with older patients in daily practice.		1		2	
…enhance my effectiveness in implementing prescribing changes during medication reviews with older patients.			1	2	
…help me to complete medication reviews with older patients more quickly in daily practice.	1	1		1	
…increase the number of medication reviews that I perform with older patients in daily practice.			1	1	1

The respondents reported that the videos would improve their performance of medication reviews with older patients (2 agree, 1 disagree), enhance their effectiveness in implementing prescribing changes (2 agree, 1 neutral) and increase the total number of medication reviews that they performed in daily practice (1 strongly agree, 1 agree, 1 neutral). Respondents reported mixed views about whether videos would help them to review older patients’ medications more quickly in daily practice (1 strongly disagree, 1 disagree, 1 agree).

In answer to the questions with free response format, respondents commented that as a resource intended to help GPs to prescribe appropriate polypharmacy for older patients, they liked that the video was short, realistic and based on a practical scenario that they would encounter in daily practice.

Practical; scenario taken from ‘daily practice’; short (study site, 1 GP2).

….short but effective, realistic (study site 2, GP1).

One respondent found it particularly helpful that the video included a monologue in which the GP outlined the underlying rationale for proposing changes to the patient’s prescription, as well as a plan for implementing the changes.

Two respondents described issues that would negatively impact on the usefulness of the video as a resource. One commented that the video ‘…even well done like this, simplifies what is a very complex interaction in practice’. The other respondent noted that time was often lacking to arrange structured consultations with patients to review their medications.

One respondent recommended a change to the video for future studies: the inclusion of an explicit summary of the recommended prescribing changes using resources such as NO TEARS [28] (a tool for conducting medication reviews) or STOPP/START [23] (validated criteria for assessing the appropriateness of prescribing in older people).

All three respondents stated that they would recommend the video to a colleague as a resource to assist the prescribing of appropriate polypharmacy for older people. One GP commented that the video created awareness of the importance of reviewing older patients’ medications to ensure that they are prescribed appropriate polypharmacy and would encourage her to do so more frequently in practice.

Highlights importance; creates awareness and I feel I will do it more frequently (study site 1, GP2).

However, another respondent noted that colleagues ‘may also feel that it doesn’t reflect ‘real-life’ with the usual time pressure we work under’ (study site 2, GP1).

#### Online system for accessing video

None of the GP respondents encountered any problems in using the online system to access the video.

GPs listed a number of additional prescribing-related resources that they would like to see included if the online system was to be further developed. These resources included a STOPP/START toolkit; printable patient information leaflets (PILs) for use in medication reviews with space for patients to write questions/problems that they could then discuss with their GPs at the next visit; patient decision aids for common conditions; and information on medication errors and interactions associated with polypharmacy in older people. One GP stated that an online forum to discuss prescribing issues with peers/pharmacists would be useful.

#### Medication review process

When asked to reflect on the last patient that they had reviewed as part of the study, all GP respondents reported making changes to the patients’ prescriptions. All prescribing changes involved the reduction or discontinuation of medications. The medications that were reportedly changed consisted of analgesics (unspecified), a proton pump inhibitor (lansoprazole), a H_2_-receptor antagonist (ranitidine), an antacid, a leukotriene antagonist (montelukast), an antihypertensive (olmesartan) and a diuretic (bendroflumethiazide).

Two GP respondents outlined issues that created difficulties in putting the reported prescribing changes into effect. For one GP, time was a barrier to implementing the prescribing changes. The other GP reported that the patient was receiving an excessive dose of a medication that was being prescribed for an unlicensed indication which was further complicated by the fact that it had been previously been initiated by another GP many years ago.

GP respondents outlined a range of issues that facilitated them in implementing the prescribing changes. All of the respondents commented on aspects of the intervention (i.e. allocated consultation time, patient engagement, consultation style of GP in the video) that facilitated the prescribing of appropriate polypharmacy. One GP also referred to concerns for patient safety and the medico-legal implications of not taking action to ensure that patients were prescribed appropriate polypharmacy.

GP respondents expressed a preference for conducting medication reviews with older patients either using scheduled consultations (*n* = 2) or on an ad hoc*/*opportunistic basis (*n* = 1). All of the respondents reported that having protected time would encourage them to perform medication reviews with older patients who are receiving polypharmacy. GP respondents listed a range of clinical issues that would also encourage them to perform medication reviews with this cohort of patients (e.g. presence of complex co-morbidities, specialist drugs, side effects). One GP commented on the need for appropriate resources in order to conduct medication reviews with these patients in practice, such as chronic disease management clinics and support from peers/community pharmacists.

### Patient feedback

All ten patient participants returned completed feedback questionnaires (response rate 100%).

#### Patients’ views on medication reviews with GPs

Most participants indicated that they would like their medication to be reviewed by their GP either once every 6 months (*n* = 4) or once every 12 months (*n* = 5). One participant stated that he wished for his medication to be reviewed by his GP every time he ordered a repeat prescription.

All participants reported that it was important to have a face-to-face consultation with their GP when having their medications reviewed.

I think it is important to review the medication and side-effects. I would find this more effective in a face-to face-appointment. Also the GP could assess the present health. Study site 2, patient 2

You can ask any questions about your medication which are bothering you and get an immediate answer with a qualified doctor. Study site 2, patient 3

…it is important to feel involved with your care. Study site 2, patient 4

#### Patient feedback on the scheduled consultation

The main outcome that patients expected from the consultation was that they would find out whether their current treatment regimens required any changes. Seven of the patients reported that the GPs recommended changes to their current prescriptions during the scheduled consultations. These patients agreed with the recommended changes, most of which involved the reduction or discontinuation of existing medications.

The patients also stated their satisfaction with the consultation. Patients welcomed the opportunity to have their medications reviewed by their GP.

I found the medication review very useful and most reassuring, as I have often wondered if all of the medication I was taking a few years ago was really necessary. Study site 2, patient 3

This was an important exercise. I now realise that without a regular review, people like me and countless others could still be taking medication they were prescribed years ago and have become ineffective…. Study site 1, patient 4

I welcomed the opportunity to discuss medication with GP as I had some worries and needed reassurance. I found the interview very satisfactory. Study site 2, patient 2

### Supplementary video coding exercise

The results of the video coding exercise are shown in Table 4. The coders agreed that the video contained ‘Modelling or demonstration of the behaviour’ as a pre-specified BCT, as well as another related BCT (‘Instruction on how to perform the behaviour’). The coders identified additional BCTs relating to the consequences of performing the behaviour (‘Information about health consequences’, ‘Information about social and environmental consequences’), in place of the pre-specified BCT (‘Salience of consequences’) and ‘Credible source’ as an additional BCT that had not been pre-specified by the research team.

**Table 4:** Outcomes of independent video coding exercise using BCT taxonomy [19]

Behaviour change techniques embedded within online video	BCTs pre-specified by research team	BCT identified by video coding team
Modelling or demonstration of the behaviour (provide an observable sample of the performance of the behaviour, directly in person or indirectly, e.g. via film or pictures for the person to aspire to or imitate)	X	X
Credible source (present verbal or visual communication from a credible source in favour of or against the behaviour)		X
Information about health consequences (provide information, e.g. written, verbal, visual, about health consequences of performing the behaviour)		X
Instruction on how to perform the behaviour (advise or agree on how to perform the behaviour)		X
Information about social and environmental consequences (provide information, e.g. written, verbal, visual, about social and environmental consequences of performing the behaviour)		X
Salience of consequences (use methods specifically designed to emphasise the consequences of performing the behaviour with the aim of making them more memorable—goes beyond informing about consequences)	X	

### Clinical record data

It was not feasible to apply the validated assessments of prescribing appropriateness (i.e. STOPP/START criteria [23], MAI [24]) to the extracted clinical data. This was because the level of detail regarding patients’ current clinical diagnoses and treatment durations in the extracted data was insufficient to determine whether patients’ clinical indications were being treated appropriately, according to the tools. Similarly, it was also not possible to conduct a complete assessment of prescribing regimen complexity using the MRCI [25] as details of additional directions for each prescribed medication were not available from patients’ medical records.

It was also not possible to verify whether any of the prescribing changes reported in the feedback questionnaires had been implemented (according to the patient case notes) as the 1 month timeline between baseline and follow-up was insufficient to detect any changes in the prescriptions that were issued to patients.

## Discussion

This study assessed the feasibility of an intervention to improve the prescribing of appropriate polypharmacy for older people in primary care. The study forms part of a systematic process of intervention development and evaluation that models the MRC framework [15]. In this way, the study serves to address limitations of previous related intervention studies whereby details of intervention development and preliminary evaluation (i.e. feasibility/pilot testing) were lacking [14].

### Intervention usability and acceptability

In assessing the primary feasibility outcomes, the feedback received from recruited GPs showed that the intervention was deemed to be both usable and acceptable for improving the prescribing of appropriate polypharmacy for older people in primary care. For example, GPs expressed largely positive views regarding the video’s content and its impact on their prescribing behaviour (i.e. increased number of medication reviews performed in daily practice, enhanced effectiveness in implementing prescribing changes). However, some reservations were expressed as to whether the clinical scenario depicted in the video truly reflected ‘real-life’ and the ‘usual time pressures’ encountered in the general practice work environment.

As detailed in our earlier intervention development work [16, 17], we recognised that the current work environment (i.e. time and resource limitations) was a major barrier to the target behaviour (i.e. prescribing of appropriate polypharmacy), and therefore, we purposefully sought to develop an intervention that would limit any additional workload for GPs in prescribing appropriate polypharmacy. The video demonstration was intended to highlight to GPs how they could potentially incorporate reviews of older patients’ medications into routine clinical practice and use available time more efficiently. Thus, the intervention was intended as a strategy for introducing small changes into GPs’ current prescribing behaviour within the constraints of the existing work environment with a view to building on these changes over time. As noted by Michie et al., ‘introducing change incrementally and building on small successes can be more effective than trying to do too much too quickly’ [29].

The scheduled consultations that formed part of the intervention approach served an important function. As noted by GP participants, protected time was an important facilitator in reviewing patients’ medications. The scheduled consultations were also endorsed by the positive feedback received from recruited patients. Patients valued the opportunity to have their medications reviewed by their GP. If the intervention is found to be effective in a future trial evaluation that is currently being developed, this component of the intervention could help contribute to the reorganisation of care required for implementing best practice guideline recommendations for patients with multimorbidity who typically require multiple medication (polypharmacy) [12]. Further refinement of the intervention for this future evaluation will involve incorporating GP participants’ suggestions to incorporate additional prescribing-related support material (e.g. validated screening tools for identifying potentially inappropriate prescribing in older people) into the online system that was used to access the video.

The study was also designed to test the feasibility of the study procedures (e.g. participant recruitment, data collection/analysis). The GP and patient recruitment strategies proved feasible whereby the sampling targets of two general practices and five older patients per practice were reached. In scaling up the number of participating general practices for future evaluations of the intervention, it will be important to engage with key stakeholders, such as GP representatives, to ensure that an appropriate level of remuneration can be offered to general practices for the time allocated to study participation. This will be dependent on the number of patients to be recruited and the number of follow-up assessments to be conducted.

### Data collection procedures

The procedures for collecting feedback from GP and patient participants were largely successful, with only one non-respondent. However, the study highlighted important limitations with the clinical data that were collected, whereby we were unable to assess patients’ clinical data using each of the validated assessment tools. We were also unable to detect any prescribing changes due to the length of the follow-up assessment (1 month post-consultation). Future evaluations of the intervention will look to include a series of follow-up assessments at six monthly intervals for a minimum of 1 year post-intervention. An assessment period with multiple assessment points will help to detect if prescribing changes are implemented and subsequently sustained. In addition, a full clinical review by a trained research pharmacist may also need to be conducted with recruited patients at baseline and follow-up. This should help to ensure that sufficient clinical information is available to allow the validated assessment tools of prescribing appropriateness to be applied (i.e. STOPP/START criteria [23], MAI [24]). The clinical review could then be coupled with pharmacy dispensing data in order to ensure that sufficiently detailed information is collected regarding medication instructions in order to allow the MRCI to be applied properly [25].

### Challenges and limitations

Despite the small-scale nature of the current feasibility study and the relatively low number of GP participants (*n* = 4), designing the study protocol posed a number of challenges. Firstly, there has, to date, been a lack of consensus in the literature as to the distinction between the terms ‘pilot study’ and ‘feasibility study’ [30, 31]. Consequently, the two terms have often been used interchangeably. A recently published conceptual framework for defining feasibility and pilot studies in preparation for randomised controlled trials will help to clarify how the terms should be applied in future research [32]. However, for the purpose of the current study, we had adopted the definition that is used by the National Institute of Health Research (NIHR) Evaluation, Trials and Studies Coordinating Centre (NETSCC) in the UK which defines feasibility studies as pieces of research that are carried out before the main study in order to estimate important parameters that are needed to design the main study and to determine whether the study can be done [33]. Hence, the study findings relating to participant recruitment and data collection procedures were more important than verifying whether reported prescribing changes had been implemented.

In collecting feedback from GP and patient participants, we chose a questionnaire-based format because time and resource limitations did not permit an in-depth follow-up study using qualitative methods (e.g. semi-structured interviews). The questionnaires included a number of free-text response options in order to allow participants to provide detailed responses. However, the availability of validated tools for constructing questionnaire items to assess the primary feasibility study outcomes (i.e. usability and acceptability) proved challenging. Therefore, items included in the GP feedback questionnaire of the online video were adapted from Davis’ *Perceived Usefulness* Scale [22]. Perceived usefulness and perceived ease of use are two constructs that form part of the TAM [22]. Empirical research indicates that the TAM is a good predictor of physicians’ behavioural intention to accept technology [34]. As a construct, perceived usefulness has been found to directly influence physicians’ intention to accept technology [34]. We did not include questionnaire items to measure *perceived ease of use* as we did not feel that the questions could be meaningfully applied to the evaluation of the online video intervention (e.g. ‘Learning to operate the online video would be easy for me.’, ‘I would find it easy to get the online video to do what I want it to do.’). In addition, there have been suggestions that the construct’s importance to the model may be reduced in target groups with a high level of competency, such as physicians [35]. Although caution has been urged in using the TAM as a predictor of technology usage outside the context in which it has been validated [36], we did not intend to make predictions based on the responses to the feedback questionnaire. The adapted questionnaire items (Tables 1 and 3) that were included in our feedback questionnaire were intended to form part of our overall assessment of the intervention’s usability and acceptability to GPs and to determine if the intervention content required additional refinement before undergoing further evaluation.

Finally, in assessing the intervention, we focussed on process measures of prescribing appropriateness using validated assessments (i.e. STOPP/START [23], MAI [24]). It must be noted that appropriate polypharmacy is an ideal concept as opposed to a fixed end-point and the threshold that differentiates between the prescribing of ‘many’ drugs and ‘too many’ drugs will vary according to an individual patient’s existing clinical conditions and life expectancy [26]. In the absence of a validated measure of appropriate polypharmacy, validated general measures of prescribing appropriateness (e.g. MAI [24], STOPP/START criteria [23]) will continue to play an important role as surrogate measures in evaluations of interventions seeking to improve prescribing for older people. In progressing to a future pilot study, it will be important to include clinical outcome measures (e.g. hospital admissions), as well as process measures. Members of the research team are currently involved in the development of a core outcome set (COS) for use in interventions aimed at improving appropriate polypharmacy in older people in primary care (http://www.comet-initiative.org/studies/details/933). This work will help in selecting the most appropriate clinical outcome measures to include in any future pilot study of the intervention.

### Specification of intervention content using BCTs

In addition to the value of the feasibility study findings, the BCT coding exercise was an important methodological step as it showed that the video intervention may have included additional BCTs to those that were specified a priori. There are a number of possible explanations to account for this finding. Firstly, the availability of formal training courses in BCT coding is a relatively recent development. It is important to note that although the BCT coders had undergone previous training in BCT coding, the training content was standardised but the delivery format varied (e.g. tutorials, online course). A number of previous BCT training courses have focused on specific subsets of BCTs (e.g. most frequently occurring BCTs) due to the practicalities involved in providing intensive training that covers all 93 BCTs within the current taxonomy [20]. Hence, our coders may not have been familiar with all of the BCTs that we had included in our intervention (e.g. ‘Salience of consequences’). Furthermore, if the coders had been specifically trained in identifying a subset of BCTs, this could have potentially resulted in a form of recall bias.

Secondly, it has been proposed that further clarification of particular BCT definitions may be required in order to improve the reliability of BCT coding [37]. A number of BCTs that were identified by the coders (e.g. ‘Information about health consequences’, ‘Information about social and environmental consequences’, ‘Demonstration of the behaviour’, ‘Instruction on how to perform the behaviour’, ‘Credible source’) have previously presented problems for other trained BCT coders with implications in terms of coding reliability (i.e. inter-rater and test-retest reliability) [37]. Based on the current findings, it is clear that in order for independent coding to perform a valid and reliable fidelity check on an intervention’s component BCTs, coders will need to have previously undergone a valid, effective and standardised training programme.

## Conclusion

A GP-targeted intervention that aimed to improve the prescribing of appropriate polypharmacy for older people in primary care that was previously developed using a systematic, theory-based approach has now undergone feasibility testing. The feasibility study findings show that the intervention was both usable and acceptable to GPs in improving the prescribing of appropriate polypharmacy for older people. Further refinements to the intervention will involve incorporating additional prescribing-related support material into the online system through which GP participants accessed the video. The feasibility study also helped to identify important limitations with the data collection procedures. Additional clinical information regarding patients’ existing conditions and the instructions they receive as to how to take their medications correctly will be required in future evaluations in order to apply validated assessment tools of prescribing appropriateness and medication regimen complexity. An additional preliminary evaluation of the intervention using a randomised pilot study is currently being developed. This will help in designing a definitive randomised trial to investigate the effectiveness of the intervention at improving appropriate polypharmacy for older people in primary care.

## Additional files

Additional file 1: GP feedback questionnaire. (DOCX 66 kb)

Additional file 2: Patient feedback questionnaire. (DOCX 50 kb)

## Abbreviations

ADE: Adverse drug event
BCT: Behaviour change technique
GP: General practitioner
MAI: Medication Appropriateness Index
MRC: Medical Research Council
MRCI: Medication Regimen Complexity Index
NICE: National Institute for Health and Care Excellence
NICRN: Northern Ireland Clinical Research Network
PIL: Patient information leaflet
START: Screening Tool to Alert doctors to Right Treatment
STOPP: Screening Tool of Older Person’s Prescriptions
SUS: System Usability Scale
TAM: Technology Acceptance Model
TDF: Theoretical Domains Framework
