# Supplementary material for: A feasibility study of a theory-based intervention to improve appropriate polypharmacy for older people in primary care
Source: Pilot Feasibility Stud. 2017 Jul 20;4:23. doi: 10.1186/s40814-017-0166-3 (PMC5520366; doi:10.1186/s40814-017-0166-3)
Supplement: Supplementary file 1 — GP feedback questionnaire. (DOCX 66 kb) [file 40814_2017_166_MOESM1_ESM.docx]

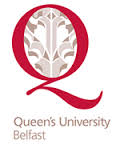


Please complete this feedback questionnaire directly AFTER you have performed medication reviews with patients who have been recruited into the study from your practice.

**Section 1 - Demographics**

1. **Are you?** ❒ Female ❒ Male
2. **How many years have you been practising as a GP?** ______ year(s).

**Section 2 – Video use and Usefulness**

1. **How many times did you watch the video before performing your first medication review with an older patient as part of this study?** ______ time(s).
2. **Please indicate your level of agreement with the following statements by circling the appropriate number.**

| *Strongly*  *disagree* |  | *Strongly*  *agree* |
| --- | --- | --- |

**Using videos like this would…**

| 1. … make it easier for me to perform medication reviews with older patients in daily practice. |  | 1 | 2 | 3 | 4 | 5 |
| --- | --- | --- | --- | --- | --- | --- |
| 1. … improve my performance of medication reviews with older patients in daily practice. |  | 1 | 2 | 3 | 4 | 5 |
| 1. …enhance my effectiveness in implementing prescribing changes during medication reviews with older patients. |  | 1 | 2 | 3 | 4 | 5 |
| 1. …help me to complete medication reviews with older patients more quickly in daily practice. |  | 1 | 2 | 3 | 4 | 5 |
| 1. …increase the number of medication reviews that I perform with older patients in daily practice. |  | 1 | 2 | 3 | 4 | 5 |

1. **As a resource intended to help you to prescribe appropriate polypharmacy for older patients...**
   1. **...what, if anything, did you like about the video?** (Please briefly outline)
   2. **...what, if anything, did you dislike about the video?** (Please briefly outline)
   3. **...would you recommend any changes to the video for future studies?**

| ❒ Yes | ❒ No | ❒ Don’t know |
| --- | --- | --- |

If YES, please briefly outline:

1. **Would you recommend the video to a colleague as a resource to help you to prescribe appropriate polypharmacy for older patients?**

❒ Yes ❒ No ❒ Unsure

Please briefly outline your response:

**Section 3 – online system for accessing video**

The questions in this section relate to the online system that you used to access the video.

1. **Did you experience any problems using the online system?**

| ❒ Yes | ❒ No |  |
| --- | --- | --- |

If YES, please briefly outline:

1. **If the online system was to be further developed as a resource to assist GPs in prescribing appropriate polypharmacy for older people, what types of additional material and/or resources would you like to see included?** (Please briefly outline)

**Section 4 – Medication review process**

1. **Think back to a medication review that you performed with an older patient as part of this study. Did you make any changes to the patient’s prescription?**

❒ Yes (Please also answer Questions 9(a), (b) and (c) below)

❒ No (Please briefly explain)

**______________________________________________________________________________**

**If you answered YES to Question 9 above:**

**______________________________________________________________________________**

**9.**

- 1. **Please specify the change(s) that you made to the patient’s prescription.**
  2. **Was there** **anything that made it difficult for you in putting this change(s) into effect?**

Please briefly outline your response:

- 1. **What helped you in putting this change(s) into effect?**

Please briefly outline your response:

1. **Please select ONE of the following statements which best describes your preference for conducting medication reviews in daily practice with older patients who are receiving polypharmacy:**

❒ I prefer to conduct medication reviews on an *ad hoc*/opportunistic basis during consultations with older patients.

❒ I prefer to conduct medication reviews using consultations that have been specifically allocated to performing medication reviews with older patients.

❒ I prefer to conduct medication reviews outside of normal consultation hours when the patient is not in front of me.

❒ I have no preference for conducting medication reviews with older patients.

❒ Other (Please briefly outline)

1. **What would encourage you to perform medication reviews with older patients who are receiving polypharmacy (≥4 regular medicines)?** (Please briefly outline your response)

**additional comments**

**Thank you for all the time and support that you have given to this project.**

Please return the completed questionnaire in the attached envelope to:

**FREEPOST, Dr. Cathal Cadogan, School of Pharmacy,**

**Queen’s University Belfast, 97 Lisburn Road, Belfast BT9 7BL**.

(No stamp required.)

Please return the completed questionnaire in the attached envelope to:

**FREEPOST, Dr. Cathal Cadogan, School of Pharmacy, Queen’s University Belfast, 97 Lisburn Road, Belfast BT9 7BL**.

(No stamp required.)

Please return the completed questionnaire in the attached envelope to:

**FREEPOST, Dr. Cathal Cadogan, School of Pharmacy, Queen’s University Belfast, 97 Lisburn Road, Belfast BT9 7BL**.

(No stamp required.)
