# Supplementary material for: A feasibility study of a theory-based intervention to improve appropriate polypharmacy for older people in primary care
Source: Pilot Feasibility Stud. 2017 Jul 20;4:23. doi: 10.1186/s40814-017-0166-3 (PMC5520366; doi:10.1186/s40814-017-0166-3)
Supplement: Supplementary file 2 — Patient feedback questionnaire. (DOCX 50 kb) [file 40814_2017_166_MOESM2_ESM.docx]

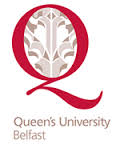


| **Instructions for completing the questionnaire** |
| --- |
| - Please complete this feedback questionnaire directly AFTER you leave your General Practitioner’s (GP’s) surgery. - Most questions involve ticking the appropriate box. - Some questions require you to write a brief response in the space provided. |

**Section 1 - Demographics**

1. **Are you?** ❒ Female ❒ Male
2. **How old are you?** ______ years.

**Section 2 – Medication reviews with your GP**

1. **Before your appointment today, when was the last time that you were aware that you had your medicines reviewed by your GP?** (Please tick ONE box only.)

❒ Within the last 6 months.

❒ Between 6 and 12 months ago.

❒ More than 12 months ago.

❒ I cannot remember the date of my last medication review.

❒ I do not know if my medicines have ever been reviewed by my GP.

1. **How often would you like your medicines to be reviewed by your GP?** (Please tick ONE box only.)

❒ Every time I order a repeat prescription.

❒ Once every 6 months.

❒ Once every 12 months.

❒ Other (please say how often you would like your medicines reviewed)

1. **Is it important for you to have a face-to-face appointment with your GP when your medicines are being reviewed?**

❒ Yes ❒ No ❒ Unsure

Please briefly explain your answer:

**Section 3 – Your appointment today**

1. **What did you hope would happen as a result of having had a medication review with your GP today?**

Please give a brief outline:

1. **Did the doctor recommend any changes to the medicines that you currently take?**

❒ Yes ❒ No

**_____________________________________________________________**

**If you answered YES to Question 7 above, please also answer Question 8 on the next page.**

**If you answered NO to Question 7 above, please add any additional comments that you would like to make on the next page.**

**_____________________________________________________________**

1. **Did you agree with the doctor’s recommended change(s) to the medicines that you currently take?**

❒ Yes ❒ No

Please briefly explain your reasons for agreeing/not agreeing with the change(s) that the doctor recommended:

**additional comments**

Please use this space if you would like to make any additional comments about the medication review that you had today with your GP.

**Thank you for the time and support that you have given to this project.**

Please return the completed questionnaire in the attached envelope to:

**FREEPOST, Dr. Cathal Cadogan, School of Pharmacy, Queen’s University Belfast, 97 Lisburn Road, Belfast BT9 7BL**.

(No stamp required.)
